# Bradycardia during Transradial Cardiac Catheterization due to Catheter Manipulation: Resolved by Catheter Removal

**DOI:** 10.1155/2017/8538149

**Published:** 2017-03-01

**Authors:** Maheedhar Gedela, Vishesh Kumar, Kashif Abbas Shaikh, Adam Stys, Tomasz Stys

**Affiliations:** ^1^Department of Internal Medicine, University of South Dakota Sanford School of Medicine, Sioux Falls, SD, USA; ^2^Sanford Cardiovascular Institute, University of South Dakota Sanford School of Medicine, Sioux Falls, SD, USA

## Abstract

*Purpose.* To report the resolution of bradycardia encountered during transradial cardiac catheterization through the catheter pullback technique in two cases.* Case Report.* A 62-year-old male and an 81-year-old male underwent coronary angiogram to evaluate for coronary artery disease and as a result of positive stress test, respectively. Upon engagement of the FL 3.5 catheter into the ascending aorta through the transradial approach, the first case developed bradycardia with a heart rate of 39 beats per minute. The second case developed profound bradycardia with a heart rate of 25 beats per minute upon insertion of the 5 Fr FL 3.5 catheter near the right brachiocephalic trunk through the right radial access.* Conclusion.* Bradycardia can be subsided by removal of the catheter during catheter manipulation in patients undergoing transradial coronary angiogram if there is a suspicion of excessive stretching of aortic arch receptors and/or carotid sinus receptors.

## 1. Introduction

Various cardiac arrhythmias can take place during cardiac catheterization. Transient bradycardia is one of the common events that can occur during cardiac catheterization. If it is prolonged, it can lead to asystole, and, ultimately, cardiovascular collapse may occur, especially in patients with severe coronary artery disease and stenosed valves. Bradycardia as a complication of cardiac catheterization is described through both the femoral and radial artery access approaches. The incidence rate of vagal reactions resulting in hypotension or bradycardia requiring atropine is 6.4% (16/250 cases) in one study of radial coronary angiograms [1]. In another study, sinus bradycardia requiring atropine occurred in 4.3% of patients (17/398) who underwent transradial coronary procedures [2]. In this article, we outline the resolution of bradycardia during cardiac catheterization through the transradial approach following catheter pullback in two cases.

## 2. Case Report

### 2.1. Case  1

A 62-year-old male with a history of hypertension, premature coronary artery disease in the family, and severe aortic insufficiency with left ventricular dilation presented with chest heaviness and shortness of breath. He was referred for a coronary angiogram and aortogram to evaluate for coronary artery disease and severe aortic insufficiency, respectively. At the beginning of the procedure, the patient's heart rate (HR) was 60 beats per minute, his blood pressure (BP) was 151/62 mmHg, and his rhythm was consistent with sinus rhythm. A 5 Fr sheath was inserted into his right radial artery. Upon engagement of the ascending aorta with an FL 3.5 catheter prior to the insertion into his left coronary artery, the patient developed sinus bradycardia, with an HR of 39 beats per minute and BP of 117/42 mmHg. Despite treatment with 0.5 mg of intravenous atropine, the patient remained at sinus bradycardia, with an HR of 36 beats per minute and BP of 96/37 mmHg. At this point, the FL 3.5 catheter was removed. The patient's HR improved, and he was recatheterized. The remaining procedure was continued safely without complications.

### 2.2. Case  2

An 81-year-old male with no significant past medical history except for dyslipidemia and hypertension underwent a coronary angiogram due to the results of his high-risk stress test. After the insertion of 5 Fr sheaths into his right radial artery, a Boston Scientific 5 Fr FL 3.5 catheter was inserted into his left coronary artery. Before the insertion, the patient's vital signs revealed sinus rhythm with an HR of 63 beats/min and BP 136/80 mmHg. Upon insertion of the catheter near the right brachiocephalic trunk prior to the engagement of his left coronary artery, profound bradycardia was noted, with an HR of 25 beats/min. The bradycardia resolved upon removal of the catheter. The remaining procedure was continued after reinsertion of the catheter safely without any consequences.

## 3. Discussion

Bradycardia is a common problem observed during the cardiac catheterization, either radial or femoral route. If it is not intervened, it may lead to asystole and hemodynamic compromise, particularly in ischemic heart disease and stenotic valvular patients. Bradycardia as a manifestation of the vasovagal reactions induced by the contrast medium, pain, or anxiety is described before in few studies. In 1974, Eckberg et al. elicited bradycardia after the injection of a contrast medium into the coronary arteries, which was mediated by cholinergic reflex, a human counterpart of the Bezold-Jarish reflex in animals [3]. Later, it was suspected that bradycardia is related to the injection of high osmolar ionic contrast material into the right coronary artery [4]. Forceful coughing restores normal cardiac rhythm by clearing the contrast material and increasing coronary blood flow [4]. These vasovagal reactions usually occur in response to pain or anxiety associated with catheterization. They can be prevented by sufficient preprocedural sedation and administration of a local anesthetic agent before vascular access is obtained with the catheter. Landau et al. demonstrated an incidence rate of 3.3% (98/2,967 patients) of vasovagal reactions requiring atropine in patients undergoing cardiac catheterization [5]. In this study, 83.7% of vasovagal episodes (82/98) occurred when vascular access was being achieved, and the remaining 16.3% of episodes occurred while the femoral arterial and venous sheaths were being removed. Proper management of a vasovagal reaction involves termination of the noxious stimulus, intravenous volume replacement, the Trendelenburg position, and administration of atropine (0.6 to 1 mg intravenously) [6]. However, in our patients, though one failed to improve despite atropine administration, bradycardia in both of these cases was alleviated by removal of the catheter.

**Figure 1:**
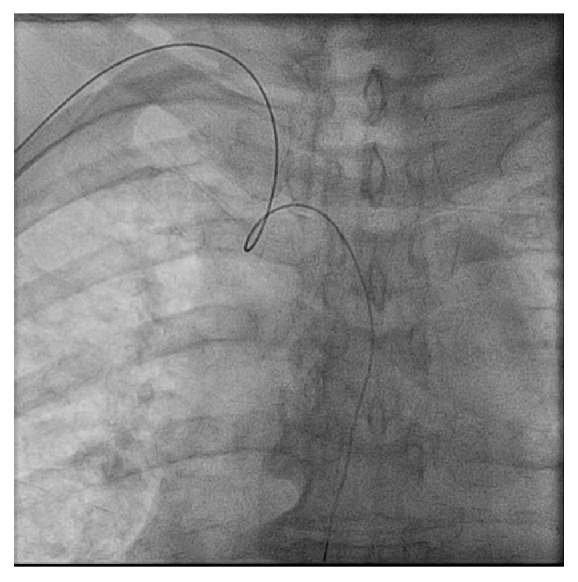
Coiling of the Guidewire at the junction of brachiocephalic artery and aorta.

**Figure 2:**
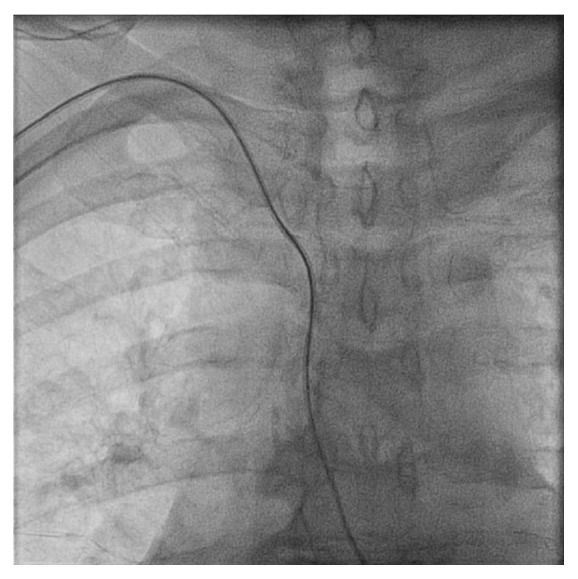
Advancement of the catheter along the Guidewire in the brachiocephalic artery and aorta.

To the best of our knowledge, transient bradycardia due to catheter manipulation has not been well described in the available literature. The bradycardia and hypotension occurred before the catheter engagement of coronary arteries in our cases which suggest these consequences are unlikely due to catheter-induced coronary spasm. When we advance the Guidewire, we may encounter coiling in the blood vessels due to the tortuosity of the vasculature (Figure 1). Subsequently, during the insertion of the catheter along the Guidewire, we may run into excessive stretching of the surrounding vasculature (Figure 2). In our cases, the bradycardia and hypotension are observed at this point. We suspect this may be due to the stimulation of aortic arch receptors and/or carotid sinus receptors due to its resolution after removal of the catheter. With the emergence of radial artery catheterization, this may become a common problem. Further investigation may be required to understand the mechanism of transient bradycardia that resolves after removal of the catheter near the brachiocephalic trunk.

## 4. Conclusion

Currently, we recommend removal of the catheter during catheter manipulation in patients undergoing radial coronary angiography to alleviate bradycardia when there is a suspicion of excessive stretching of aortic arch and/or carotid sinus receptors.
